# Role of Perinatal Inflammation in Neonatal Arterial Ischemic Stroke

**DOI:** 10.3389/fneur.2017.00612

**Published:** 2017-11-16

**Authors:** Antoine Giraud, Clémence Guiraut, Mathilde Chevin, Stéphane Chabrier, Guillaume Sébire

**Affiliations:** ^1^EA 4607 SNA EPIS, Jean Monnet University, Saint-Etienne, France; ^2^Child Neurology Division, Department of Pediatrics, McGill University, Montréal, QC, Canada; ^3^French Center for Pediatric Stroke and Pediatric Rehabilitation Unit, Department of Pediatrics, Saint-Etienne University Hospital, Saint-Etienne, France

**Keywords:** NAIS, risk factors, physiopathology, chorioamnionitis, vasculitis, immunothrombosis, treatment, neuroprotection

## Abstract

Based on the review of the literature, perinatal inflammation often induced by infection is the only consistent independent risk factor of neonatal arterial ischemic stroke (NAIS). Preclinical studies show that acute inflammatory processes take place in placenta, cerebral arterial wall of NAIS-susceptible arteries and neonatal brain. A top research priority in NAIS is to further characterize the nature and spatiotemporal features of the inflammatory processes involved in multiple levels of the pathophysiology of NAIS, to adequately design randomized control trials using targeted anti-inflammatory vasculo- and neuroprotective agents.

## Introduction

Neonatal arterial ischemic stroke (NAIS) is defined by a symptomatic arterial ischemic stroke occurring in the neonatal period, i.e., the first 28 days of life (1). NAIS has to be differentiated from the other subtypes of perinatal stroke, namely cerebral sinovenous thrombosis, neonatal hemorrhagic stroke, and arterial presumed perinatal ischemic stroke (2). Each of these entities differs from NAIS on several aspects, either: (i) affecting different vessel types each of them, such as vein, artery or capillary, featured by specific biological aspects as well as responses to stresses (3), (ii) occurring within distinct—even if somehow overlapping—developmental time frames across gestational and postnatal ages (2), (iii) having different clinical and imaging presentations and outcomes (2), or (iv) being consequently associated with distinct sets of risk factors and causes.

Neonatal arterial ischemic stroke is one of the commonest forms of pediatric stroke, causing a heavy burden of life-long motor, cognitive, and/or behavioral disabilities (2). The pathophysiology of NAIS remains largely unknown (2, 4); hence, there is no evidence-based preventive or curative vasculo- or neuroprotective strategy available for patients affected by NAIS (5).

After having reviewed the literature to assess epidemiological, clinical and fundamental data, we discuss the role of perinatal inflammation in the causal pathways leading to NAIS.

## Epidemiology of NAIS

According to the five population-based studies currently available, the range of prevalence of NAIS varies from 6 to 17/100,000 (6–11). This represents one-fourth of all perinatal strokes syndromes (6).

Based on the review of the literature, the risk factors of NAIS as defined from the seven case–control studies dedicated to NAIS – or other studies in which specific data about NAIS could be extracted—have been summarized in the Table 1 (8, 9, 12–16).

**Table 1:** Identified risk factors of NAIS.

Design (ref)	Cases (*n*)	Controls (*n*)	Significant risk factors after univariate analysis	Independent risk factors after multivariate analysis
R (15)	12	24	Assisted ventilation for resuscitation (OR 7.0; 95% CI 1.04–53.5), lower AS at 1 min (6 vs. 8, *p* < 0.05)	ND
P (13)	100	45,508	**Premature rupture of membranes (OR 3.0; 95% CI 1.1–6.8)**, male (OR 1.5; 95% CI 1.0–2.4), nulliparity (OR 1.8; 95% CI 1.1–2.7), C-section (OR 3.3; 95% CI 2.12–4.9), twin-gestation (OR 3.7; 95% CI 1.0–10), traumatic delivery (OR 9.6; 95% CI 4.8–18), ECS (OR 1.8; 95% CI 1.0–3.3), AS at 1 min ≤ 7 (OR 3.3; 95% CI 1.9–5.4), resuscitation at birth (OR 4.4; 95% CI 2.5–7.3), immediate transfer to a neonatal unit (OR 4.9; 95% CI 2.6–8.7)	ND
R (9)	49	490	Male (OR 1.8; 95% CI 1.0–3.4)	ND
R (8)	24	48	**Meconium-stained amniotic fluid (OR 6.8; 95% CI 2.3–21)**, C-section (OR 3.5; 95% CI 1.1–11), abnormal FHR (OR 5.1; 95% CI 1.5–17)	ND
R (14)	52	156	**Maternal fever (OR 7.5; 95% CI 1.5–38.7)**, **early-onset sepsis/meningitis (OR 7.0; 95% CI 1.8–27.1)**, **meconium-stained amniotic fluid (OR 4.5; 95% CI 2.1–9.8)**, nulliparity (OR 2.0; 95% CI 1.0–3.7), abnormal FHR (OR 8.2; 95% CI 3.5–19.1), ECS (OR 18.0; 95% CI 5.3–61.1), AS at 1 min ≤ 3 (OR 45.0; 95% CI 5.9–340.7), AS at 5 min < 7 (OR 19.5; 95% CI 4.4–86.4), arterial UC pH < 7.10 (OR 14.9; 95% CI 3.4–66.1), hypoglycemia < 2.0 mmol/l (OR 11.0; 95% CI 3.7–33.3)	**Maternal fever (OR 10.2; 95% CI 1.3–78.5), early-onset sepsis/meningitis (OR 5.8; 95% CI 1.1–31.9)**, AS at 5 min < 7 (OR 18.1; 95% CI 3.4–96.8), hypoglycemia < 2.0 mmol/l (OR 13.0; 95% CI 3.2–52.6)
R (12)	37	223,547	ND	**Maternal fever (OR 4.0; 95% CI 1.2–13.7), congenital infection (OR 14.2; 95% CI 2.8–71.0), neonatal infection (OR 9.5; 95% CI 4.4–20.7)**, maternal hypertension (OR 2.8; 95% CI 1.5–5.2), child birth trauma (OR 8.7; 95% CI 4.2–18.0), child birth asphyxia (OR 22.2; 95% CI 7.0–70.9), hematological diseases^a^
P (16)	79	239	**Viral infection during gestation (OR 3.2; 95% CI 1.2–8.4)**, **prolonged rupture of membranes (OR 11.5; 95% CI 4.0–33.1)**, **maternal fever (OR 5.3; 95% CI 2.0–14.3)**, **thick meconium (OR 4.6; 95% CI 2.2–9.3)**, family history of seizures (OR 5.5; 95% CI 2.1–14.0), family history of neurologic diseases (OR 6.6; 95% CI 2.4–18.7), maternal AI disease (OR 15.7; 95% CI 1.8–137), gynecological problems (OR 5.3; 95% CI 1.5–18.6), nulliparity (OR 3.1; 95% CI 1.7–5.5), abdominal pain during gestation (OR 60; 95% CI 3.4–1,044), prolonged second stage (OR 3.7; 95% CI 1.8–7.3), tight nuchal cord (OR 2.6; 95% CI 1.2–5.9), abnormal FHR (OR 7.3; 95% CI 3.9–13.7), assisted vaginal delivery (OR 1.8; 95% CI 1.1–3.2), failed instrumental delivery (OR 12.6; 95% CI 4.0–39.6), ECS (OR 6.8; 95% CI 3.8–12.5), AS at 1 min < 3 (OR 49.8; 95% CI 2.8–883.7), AS at 5 min < 5 (OR 35.7; 95% CI 2.0–653.6), arterial UC pH < 7.10 (OR 41.9; 95% CI 5.2–333), major neonatal resuscitation (OR 18.5; 95% CI 4.0–85.6), GA > 42 weeks (OR 21.5; 95% CI 1.1–420), male (OR 2.2; 95% CI 1.3–3.8), IUGR (OR 3.9; 95% CI 1.0–15.1)	One *intrapartum* factor among the following (namely: **prolonged rupture of membranes**, **maternal fever**, **thick meconium**, prolonged secnd stage, tight nuchal cord, abnormal FHR) vs. none of the above factors (OR 5.9; 95% CI 1.9–18.0), male (OR 2.8; 95% CI 1.2–7.0), family history of seizures (OR 6.6; 95% CI 1.7–25.6)

*Summary of the seven case–control studies published between 1997 and 2016*.

*^a^Hematological diseases: child sickle cell disease (OR 11.4; 95% CI 3.3–39.4), child sickle cell trait (OR 6.9; 95% CI 1.7–28.1), and child constitutive thrombophilia (OR 413.2; 95% CI 111.1– > 1,000)*.

*AI, autoimmune; AS, Apgar score; CI, confidence interval; ECS, emergency C-section; FHR, fetal heart rate; GA, gestational age; IUGR, intrauterine growth restriction; ND, not done; OR, odds ratio; P, prospective; R, retrospective; Ref, reference; UC, umbilical cord*.

*Inflammatory risk factors are in bold type*.

Inflammatory markers are found as risk factors of NAIS in all case–control studies in which they were studied (8,12–14,16) (Table 1). Several direct markers of active perinatal infection/inflammation are independent risk factors of NAIS, namely maternal fever [odds ratio (OR): 4.0–10.2, in the three studies including multivariate analysis (12, 14, 16)], and neonatal infection [OR: 5.8–9.5, in two of these studies (12, 14)]. The only other independent and consistent across studies risk factor of NAIS is *peripartum* asphyxia (12, 16). *Peripartum* asphyxia can either be secondary to infection and subsequent inflammation triggered by systemic exposure to pathogen-associated molecular patterns (PAMPs), or be a powerful inducer of sterile inflammation *via* the systemic or intracerebral release of damage-associated molecular patterns (DAMP) (cf. see Primary Phase of Neonatal Arterial Ischemic Brain Injury) (17).

Materno-fetal and postnatal inflammation is mostly caused by infection. Neonatal bacterial meningitis is classically complicated by arterial ischemic stroke due to focal arteritis (14, 18–22). Histological chorioamnionitis has not been studied as a possible risk factor (8, 9, 12–16). However, it is well possible that most mothers displaying fever suffer of clinical chorioamnionitis. Further investigations of the potential association between chorioamnionitis and NAIS need to be performed.

Few non-infectious/inflammatory features are associated with NAIS. Male sex was found as an independent risk factor in only one of the seven case–control studies (16) (Table 1). Genetic prothrombotic risk factors are not associated with NAIS occurrence. The only study which identified thrombophilia from genetic origin as an independent risk factor of neonatal ischemic stroke was based on a heterogeneous cohort of term, late preterm, and early preterm newborns (12). Several other studies assessed the association between constitutive prothrombotic risk factors and NAIS, with contradictory findings (23–25). The one with the most reliable methodology found a similar rate of thrombophilia at 12 months between the NAIS and the control groups (23). All these studies only investigated constitutive/genetic coagulation markers. To our knowledge, no controlled study was performed in close temporal relationship with the NAIS to assess the expected acute activation of prothrombotic factors.

In sum, perinatal infection/inflammation is the only independent risk factor of NAIS consistently reported up to now. Genetic prothrombotic risk factors do not appear to be associated with NAIS occurrence.

## Physiopathology of the Arterial Occlusion Leading to NAIS

### Role of Inflammation in the Disruption of the Cerebral Arterial Blood Flow in NAIS

Given the tight reciprocal activation between inflammatory and coagulation cascades, it is quite possible that inflammation promotes thrombus formation within placental, umbilical cord or other vessels feeding the cerebral blood flow. According to a classic pathophysiological hypothesis of NAIS, such thrombus would then migrate and occlude cerebral arteries leading to embolic stroke (26). This embolic hypothesis is also supported by the preponderant distribution of NAIS in the middle cerebral arterial territories, and in few instances by the detection of thrombotic/embolic events proximal or distal to the NAIS (27).

However, this embolic hypothesis is challenged by: (i) the imbalanced distribution of NAIS between the anterior *versus* posterior intracranial arterial territories even when the asymmetry of anterior *versus* posterior blood flows is taken into account (13, 28, 29); (ii) the infrequent occurrence of extracerebral infarcts concomitant to NAIS (13, 28, 29); and (iii) angiographic findings from newborns with NAIS showing that 22–65% of them present focal disruptions of the anterior circulation which, in certain cases might correspond to arterial wall defects, or to thrombi generated *in situ* from an inflamed arterial wall (28, 29). Based on these elements, we hypothesized that maternofetal inflammation induces a focal arteritis specifically affecting NAIS susceptible cerebral arteries, namely the middle cerebral artery (MCA), anterior carotid artery and intracranial internal carotid artery (13, 28, 29).

Using a preclinical rat model of chorioamnionitis induced by pathogen components [lipopolysaccharide (LPS) from *Escherichia coli*], we showed that end-gestational inflammation combined with a classic prothrombotic stress (transcutaneous laser exposure of the artery of interest), but not sole prothrombotic stress, targeting the MCA triggers NAIS (30). On one hand, the walls from neonatal cerebral arteries susceptible to stroke displayed a constitutively higher expression of proinflammatory cytokines in NAIS-susceptible *versus* non-susceptible arteries. On the other hand, pups from LPS-exposed dams presented a cerebral arteritis characterized by an increased number of inflammatory cells and expression of proinflammatory cytokines [interleukin (IL)-1/IL-1 receptor antagonist (IL-1Ra) ratio] within NAIS-susceptible, but not non-susceptible, arteries (30).

These preclinical results support, beside the embolic hypothesis, the contribution of a focal arteritis and thrombosis in the pathophysiology of NAIS.

### Links between Perinatal Inflammation and Thrombosis within NAIS Susceptible Arteries

#### Established Mechanistic Links between Systemic Inflammation and Thrombosis

Inflammation has been well characterized as a potent procoagulant phenomenon.

**Figure 1:**
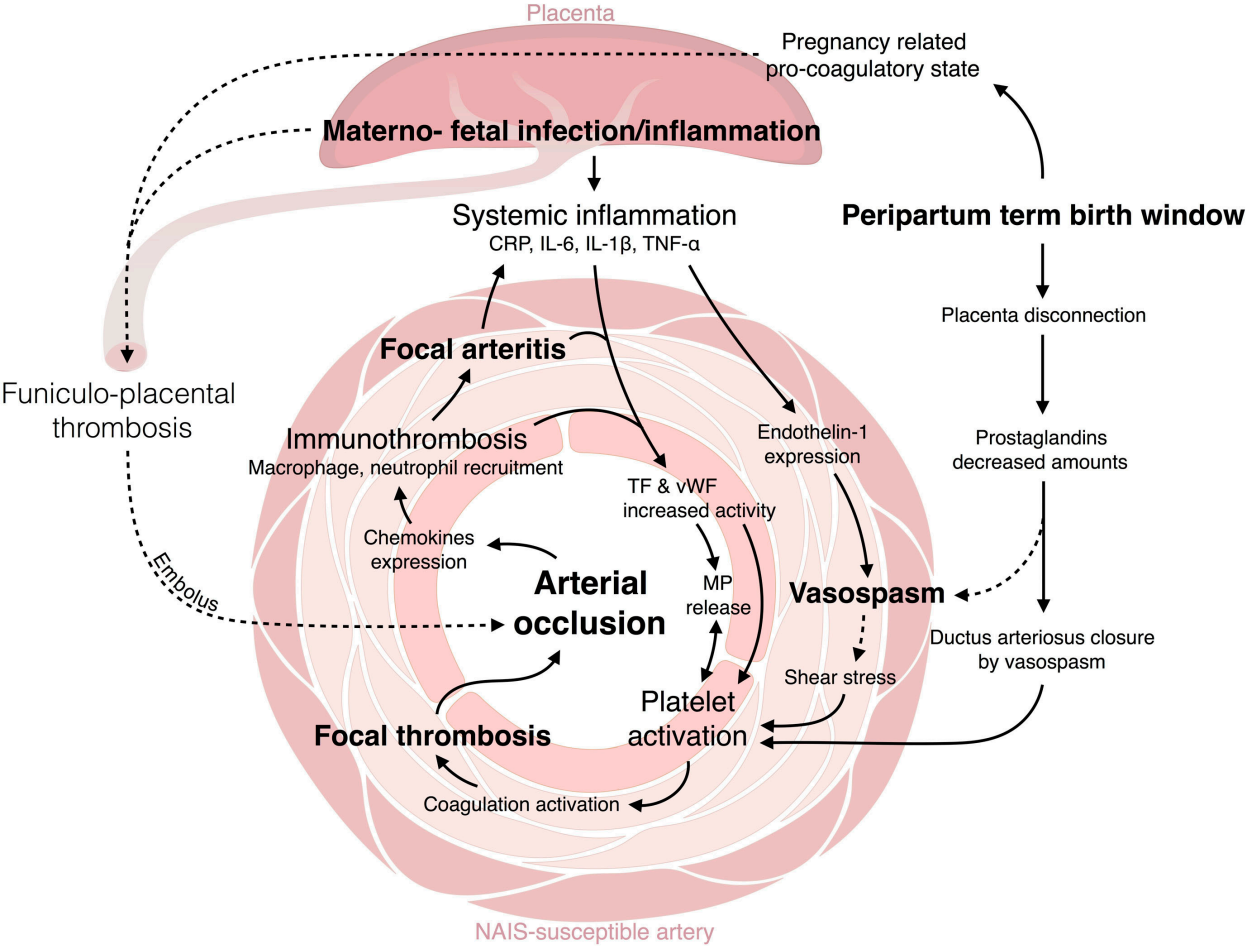
Crosstalk between inflammation and thrombosis. C-reactive protein (CRP) and proinflammatory cytokines [interleukin (IL-β), IL-6, tumor necrosis factor (TNF)-α] are mediators implicated in the materno-fetal inflammation (31–33). CRP increases tissue factor (TF) activity *in vivo* (34). TNF-α induces the endothelium activation: the production of TF and release of von Willebrand factor (vWF)-propeptide (35, 36). Microparticles (MPs), released from platelets, macrophages, and endothelial cells upon activation (37), trigger coagulation cascades *via* TF and vWF binding sites (37). Activated mononuclear cells attract and activate platelets *via* TF inducible expression (36–38). Monocyte activation leads to TF-dependent thrombin generation and activation of coagulation (38, 39). Neutrophil extracellular traps (NET) regulate both inflammation and coagulation (40). Proinflammatory cytokines and activated monocyte/macrophages are present within the wall of NAIS-susceptible arteries (30). Glycosaminoglycan synthesis and anticoagulant activity is decreased under inflammation through TF pathway inhibitor and antithrombin interactions with their serine proteinases is impaired (37, 39, 41). Antithrombin activity is downregulated due to its consumption to counteract the mononuclear cells activation and thrombin generation (37). Thrombomodulin is downregulated upon TNF-α exposure (37, 39). IL-1β and TNF-α contribute to vasoconstriction *via* the upregulation of endothelin-1 (42–44). Endothelin-1 increases superoxide anion production, cytokine release (45), and induces prothrombotic effect close to the endothelin-1-induced vasospasm. The *ductus arteriosus* closure is triggered by a vasospasm caused by the decreased plasma concentrations in PGE2 and the increased O_2_ tension (46, 47). This mechanism could also happen in NAIS-susceptible fetal cerebral arteries. The thrombotic process is associated with the recruitment at the coagulation site of innate immune cells, this is the immunothrombosis. Thrombin induces the expression of proinflammatory cytokines and chemokines by the endothelial cells (37). Platelets are also involved in the trapping and clearing of bacterial agents (40, 48, 49). Activated platelets support neutrophils and monocytes recruitment (e.g., through CXCL1, CXCL4, CXCL5, CCL3, CCL5, CCL7 expression), adhesion (e.g., platelet P-selectin, CD40 ligand expression), and activation [e.g., receptor expressed on myeloid cell (TREM)-1 induced proinflammatory activity] (36, 37, 40).

The C-reactive protein (CRP) and proinflammatory cytokines, including IL-1β, IL-6, and tumor necrosis factor (TNF)-α, are well-established mediators which are implicated in the materno-fetal inflammation, combining maternal immune activation and/or fetal inflammatory response syndrome (FIRS) (31–33) (Figure 1). CRP is known to increase the tissue factor (TF) activity *in vivo* (34). Exposure of endothelial cells to proinflammatory cytokines, such as TNF-α, induces the endothelium activation, its production of TF and release of von Willebrand factor (vWF)-propeptide, which are interacting key determinants of platelet activation and aggregation (35, 36) (Figure 1). Microparticles (MPs) are known to: (i) be produced by a large variety of activated cells, and (ii) be released from platelets, macrophages and endothelial cells during bacterial infection (37). MPs carry on their surface a range of molecules implicated in triggering coagulation cascades *via* TF and vWF binding sites (37). Activated mononuclear cells (upon bacterial infection through TNF-α or IL-6 exposure) are able to attract and activate platelets *via* TF inducible expression (36–38) (Figure 1). Cytokine-mediated monocyte activation leads to TF-dependent thrombin generation and activation of coagulation (38, 39). Some structures, such as neutrophil extracellular traps (NET) are involved in the regulation of both inflammation and coagulation (40). Such cellular and molecular processes bridge the activation of coagulation with data from a preclinical model of NAIS showing that proinflammatory cytokines and activated monocyte/macrophages are present within the wall of NAIS-susceptible arteries from pups exposed to perinatal inflammation (30). Under inflammation, procoagulant proteins are not the only ones to be affected: natural anticoagulant mechanisms are also downregulated. For instance, the glycosaminoglycan synthesis is downregulated on the endothelial surface under inflammation and its anticoagulant activity through TF pathway inhibitor and antithrombin interactions with their serine proteinases is impaired (37, 39, 41). Antithrombin activity is downregulated due to its consumption to counteract the mononuclear cells activation and thrombin generation (37). Another anticoagulant protein, thrombomodulin, which acts by inhibiting the thrombin procoagulant activity, has been shown to be downregulated upon TNF-α exposure (37, 39).

Altogether, clinical data and preclinical modeling show that inflammatory processes happening within placenta, systemic circulation, and wall of NAIS-susceptible arteries are part of the causal pathway of NAIS.

#### Contribution of Systemic Inflammation to Vasoconstriction

Proinflammatory cytokines such as IL-1β and TNF-α have been shown, using endothelial cell culture and MCA occlusion models, to contribute to vasoconstriction *via* the upregulation of endothelin-1 (42–44). Endothelin-1 plasma levels are increased during endotoxemia (45). Endothelin-1 is also known to be able to increase superoxide anion production, cytokine release (45), and to induce subsequent prothrombotic effects adjacent to the endothelin-1-induced vasospasm. According to this mechanism, vasoconstriction might be a key factor in NAIS pathophysiology.

Only few hours after birth, the *ductus arteriosus* remodeling occurs. This remodeling is provoked by the decreased newborn’s plasma concentrations in prostaglandin E2 (PGE2; placental production) and the increased dioxygen (O_2_) tension. This combination triggers a vasoconstriction (46, 47) and platelets are recruited during this process (50). These two mechanisms could act similarly on the fetal cerebral arteries and trigger a focal vasospasm (Figure 1).

Premature babies are more often affected by chorioamnionitis than term babies; however, they are less affected by NAIS (51, 52). This observation might be seen as challenging the inflammatory hypothesis of NAIS, which happens mostly in term babies. However, the inflammatory response of preterm newborns is immature and weaker compared to the one from term newborns (53, 54). In addition, premature babies have immature baroreflex which might prevent the occurrence of vasospasm and subsequent ischemia (52, 55–57). These elements might contribute to prevent the occurrence of NAIS in preemies.

#### Contribution of Thrombosis to Inflammation

Inflammation and coagulation are tightly interrelated processes in bidirectional ways. For instance, thrombin, which is a potent activator of the coagulation cascade, is also able to induce the expression of proinflammatory cytokines and chemokines by the endothelial cells (37). Moreover, platelets are not only involved in coagulation in order to protect the host from arterial wall damage but also involved in the trapping and clearing of bacterial agents [e.g., *E. coli* binding through Toll-like receptor (TLR) 4 and cluster of differentiation (CD) 62 (48)] (40, 49). Consequently, the thrombotic process is associated with the recruitment at the coagulation site of innate immune cells, the so-called immunothrombotic process. Activated platelets have an important role in immunothrombosis; they have been shown to support neutrophil and monocyte recruitment [e.g., through chemokines C-X-C chemokine ligand (CXCL) 1, CXCL4, CXCL5, C-C chemokine ligand (CCL) 3, CCL5, CCL7 expression], adhesion (e.g., platelet P-selectin, CD40 ligand expression), and activation (e.g., triggering receptor expressed on myeloid cells (TREM)-1 induced proinflammatory activity) (36, 37, 40). These immune cells could induce a deleterious inflammation within the wall of a thrombosed artery and trigger further occlusion and thrombosis as observed in disseminated intravascular coagulation subsequent to sepsis (36, 37, 39, 40).

Hence, immunothrombotic processes occurring within the lumen and the resulting inflammation of the adjacent wall of NAIS-susceptible arteries might be part of a vicious circle driving the pathophysiology of NAIS, even in the frame of a primary embolic etiopathogenic process triggering arterial wall inflammation (40).

## Inflammatory Pathways Involved in Brain Ischemic Injuries of the Term Newborn

Based on the above clinical and preclinical findings, NAIS results from a multiple hit mechanism combining perinatal inflammation and hypoxia–ischemia (HI). In sharp contrast to pre-natal HI or postnatal inflammation happening at least 24 h before HI, which have been shown in preclinical models to be neuroprotective (the so-called preconditioning effect) (58–60), prenatal inflammation sensitizes the brain to immediately postnatal HI injuries (61–63). In newborns, such pre-natal inflammatory exposure (e.g., due to chorioamnionitis) might be involved in aggravating the HI injury due to NAIS. The inflammatory pathways involved in HI- and infection/inflammation plus HI-induced NAIS are summarized below.

### Primary Phase of Neonatal Arterial Ischemic Brain Injury

**Figure 2:**
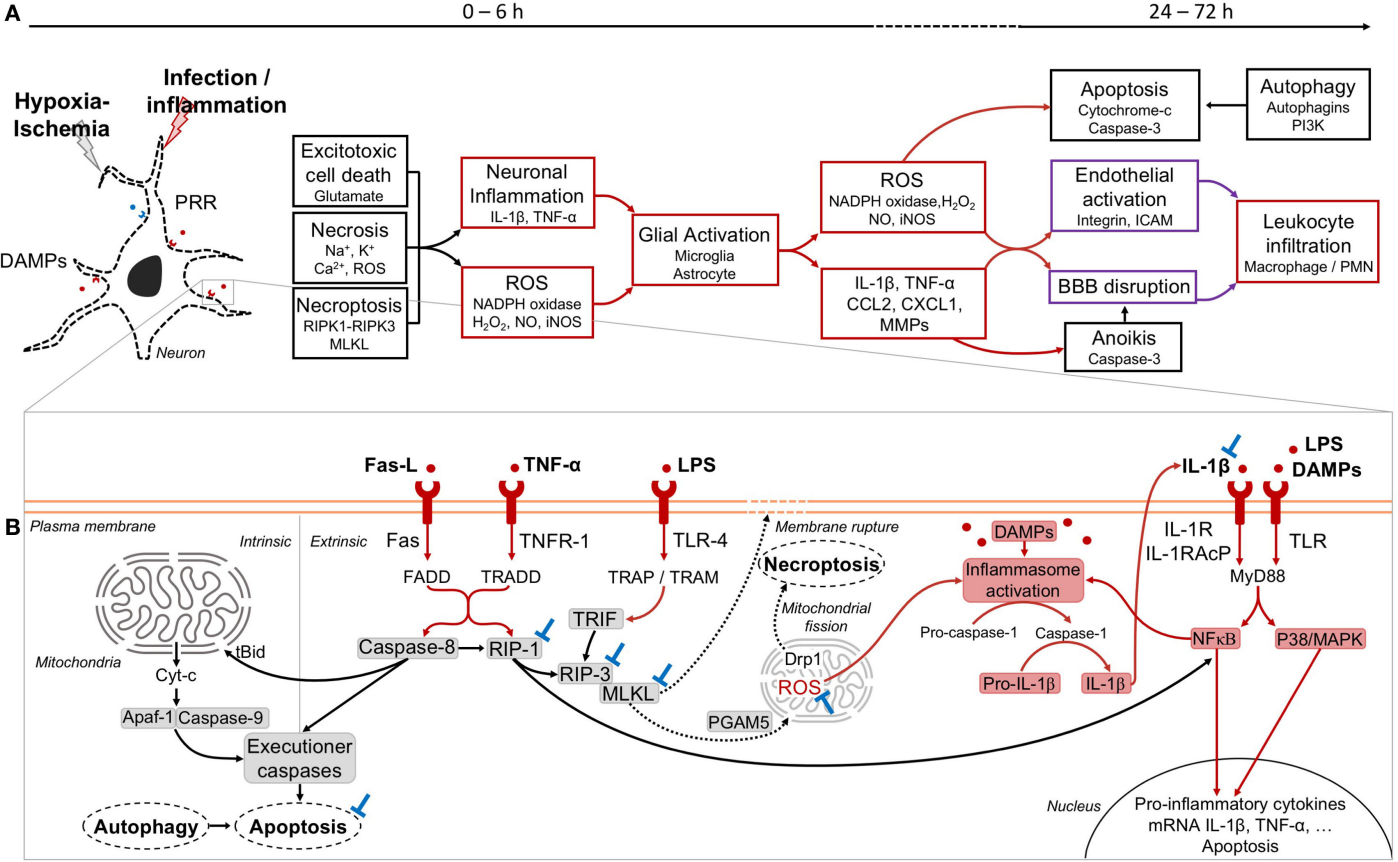
Phases of injury occurring in neonatal arterial ischemic stroke (NAIS) and mechanistic pathways. **(A)** The first phase of injury in NAIS occurs between 0 and 6 h after the exposition to hypoxia–ischemia (HI) alone or infection/inflammation plus HI. This phase is characterized by different cell death types, including excitatory cell death, necrosis, and necroptosis. These primary cell deaths will induce several inflammatory cascades. Exposure to lipopolysaccharide (LPS) + HI releases DAMPs within neurons leading to an overexpression of IL-1β through inflammasome activation, which also leads to nuclear factor-κB (NFκB)-induced tumor necrosis factor (TNF)-α synthesis (73, 74). This will further result in the activation of the glial cells and the increase of the inflammation through the release of reactive oxygen species (ROS) and several inflammatory molecules by these cells. The secondary phase occurs between 24 and 72 h after NAIS and includes apoptosis, anoikis, and autophagy cell deaths. Overall, this will induce the activation of the endothelium of the brain vessels and can lead to the rupture of the blood–brain barrier (BBB) and the infiltration of leukocytes within the brain. **(B)** Cell death and inflammatory pathways at play within a neuron in the injured brain. Extrinsic apoptosis is induced by inflammatory molecules, such as Fas ligand (Fas-L) and TNF-α and further activation of their respective receptors FAS and TNFR-1. This leads to the recruitment of caspase-8. The activation of caspase-8 induces the recruitment of executioner caspases and subsequent cell death by apoptosis (75, 76). Activated caspase-8 negatively regulates necroptosis signaling by cleaving receptor interacting protein kinase (RIP)-1 (69, 77). In intrinsic apoptosis, caspase-8 can recruit and activate proapoptotic proteins, including Bax and Bak through the activation of t-Bid. The excess of proapoptotic protein as compared to antiapoptotic protein (Bcl2, Bcl-xL) results in an opening of the mitochondrial permeability transition pore, and the release of cytochrome-c (Cyt-c) into the cytoplasm. This leads to the apoptosome formation with the recruitment of apoptotic protease activating factor-1 (Apaf-1) and caspase-9, and the induction of the cell death by apoptosis. Necroptosis is induced by different signaling pathways, including Fas-L–Fas, TNF-α–TNFR-1, and LPS–TLR-4. This will induce the dimerization of RIP-1 and RIP-3, thus inducing the phosphorylation of RIP-3 (78). In the TLR-4-induced necroptosis, RIP-3 and MLKL are activated, but without RIP-1. Instead of RIP-1, TIR-domain-containing adapter-inducing interferon-β (TRIF) will associate with RIP-3 and subsequently induce necroptosis (79, 80). Upon RIP-3 activation, MLKL is recruited, phosphorylated, and translocated to the plasma membrane to initiate cell death through the disruption of the membrane integrity (80). Phosphorylated MLKL can also interact with the mitochondrial phosphatase PGAM5 and further induced ROS expression, and may activate dynamin-related protein 1 (Drp1) that could ultimately lead to cell death through mitochondrial fission (75, 80, 81). The release of mitochondrial ROS within the cytoplasm of the neuron, as well as the DAMPs can induce the activation of the inflammasome (82, 83). The activation of caspase-1 will induce the cleavage of the pro-IL-1β into IL-1β. The inflammation will pursue with the autocrine-paracrine loop of IL-1β activation and the activation of transcription factors, such as NFκB and P38/MAPK (83). Potential blocking agents (⊥) are as following: IL-1 blockers (e.g., IL-1Ra) (74), necrostatin-1 for RIP-1 (67), GSK’872 for RIP-3 (84), necrosulfonamide for MLKL (85), caspase inhibitors for apoptosis (86–88), and apocynin for nicotinamide adenine dinucleotide phosphate (NADPH) oxidase targeting (89). Color codes: black: cell death; red: inflammation; purple: vascularization; blue: blockades.

The neural cell stress associated with NAIS is mainly due to the combination of energy failure, excess of intracellular Ca^2+^, and glutamate release, as well as ionic imbalance and oxidative stress (64–66). All these pathways are key mechanisms driving neural cell death (64, 66–68). We learned from a preclinical model of NAIS, that excitotoxic cell death, necrosis and programmed necrosis (necroptosis) occur between 0 and 6 h after exposures to sole HI or pathogen components plus HI (Figure 2A) (66, 69–72).

Pattern recognition receptors (PRR) such as TLR recognize pathogens as well as DAMP (73, 74, 90–94). Inflammation sensitizes the neonatal brain to subsequent HI injury (54, 73, 74, 95). For instance, Stridh et al. showed in a newborn mouse model [at postnatal day (P) 9, i.e., a level of brain development equivalent to the term human newborn] of NAIS that TLR-2 deficiency protected the brain from infarcts (94). Energy failure due to hypoxia and inflammation combine their effects to increase the oxidative stress (96, 97). Besides, LPS and other pathogen components interact with various TLR to increase the synthesis of a wide set of proinflammatory cytokines and chemokines (Figure 2B) (54, 73, 74, 95). For instance, in a preclinical model of NAIS, exposure to LPS plus HI leads to an autocrine/paracrine loop of neuronal self-injury, mediated by inflammatory molecules: IL-1β, TNF-α, reactive oxygen species (ROS) production, and mitogen-activated protein kinases (MAPK)-induced apoptosis (Figure 2B) (73, 74). LPS combined with HI leads to glial activation and neurotoxic molecule release such as IL-1β-induced matrix metalloproteinase (MMP)-9, nitric oxide (NO), and inducible NO synthase (iNOS) (66, 73). In these LPS plus HI-exposed brains, NO and MMP-9 combine their effects to alter the blood brain barrier (BBB) by degrading the lamina of intracerebral blood vessels (67, 74). Such disruption enables proinflammatory and/or neurotoxic mediators to leak through the BBB, and thus increasing the extent of NAIS in preclinical models (74, 98) (Figure 2A).

Necroptosis is an early cell death pathway which is triggered by inflammatory mediators led by TNF-α and TNF family death receptor (TNFR): TNFR-1, FAS, and TLR, namely TLR-3 and TLR-4 (75, 79, 84, 99). The necrosome is a complex that requires the presence of activated receptor interacting protein kinase (RIP)-1, RIP-3 and the pseudo-kinase mixed lineage kinase domain-like (MLKL) to execute necroptosis (75, 99) (Figure 2B). All the mechanisms by which MLKL induces cell death are not totally elucidated (75, 81, 99). It was shown in a preclinical model that cerebral expression of TNF-α was triggered by LPS plus HI exposure (73). Accordingly, in this NAIS model RIP-3 expression was increased after LPS plus HI (74). IL-1β-induced MMP-9 can also activate necroptosis *via* Fas-ligand (Fas-L) interaction through Fas-associated death domain (FADD) (70, 96, 99). In line with these findings, it was shown that necrostatin-1—a RIP-1 inhibitor—administered after HI injury in P7 mice was neuroprotective: this inhibitor is able to prevent forebrain injury, as well as attenuates oxidative stress and mitochondrial dysfunction (69, 71). However, since RIP-1 is an important crosstalk molecule between apoptosis and necroptosis pathway, it has been observed in neonatal HI model that RIP-1 blockade increased apoptotic cell death (69).

### Secondary Phase of Neonatal Arterial Ischemic Brain Injury

The secondary phase of ischemic injury occurs between 24 and 72 h after NAIS and implicates apoptosis, anoikis and autophagy cell deaths. Intrinsic and extrinsic forms of apoptosis are known to be involved in NAIS due to pure HI or HI combined to pathogen exposure (67, 68, 72) (Figure 2B). Many preclinical studies characterized the involvement of apoptotic cell death in the genesis of NAIS and provided evidence in favor of neuroprotective strategies targeting apoptotic pathways (86–88, 100–102).

It is well known that apoptotic and autophagic cell death pathways crosstalk, and that autophagy can block apoptosis by sequestration of mitochondria (68) (Figure 2B). The induction of autophagy just after neonatal HI may be a neuroprotective mechanism by limiting apoptosis (68, 103). On the other hand, autophagy seems to be implicated in HI-induced cell death (104). Besides, another form of apoptosis triggered by cell detachment from the extracellular matrix—namely anoikis—could follow HI and/or inflammation exposures. Anoikis is induced by increased MMPs, including MMP-9 and activation of Fas receptor, which initiates the apoptosis cascade (105). This cell death pathway was assessed by our group: we showed an overexpression of MMP9 after HI plus inflammation injury (73, 74). Besides, the use of an MMP-9 competitive inhibitor shrank the size of LPS plus HI-induced brain infarcts (73, 74) (Figure 2A). To our knowledge, this is the first demonstration of anoikis-induced cell death in a model of combined inflammation and/or HI.

## New Hypotheses Bringing New Treatments

Given that the diagnosis of NAIS is often delayed due, in most cases, to the prenatal onset and/or to the absence, or paucity and diagnostic delays, of neonatal symptoms, future therapies should focus on the control of preinsult determinants most often acting prenatally—e.g., through anti-inflammatory intervention, such as IL-1Ra, or on postinsult neonatal mechanisms—e.g., through hypothermia therapy (HT)—rather than on less feasible per-insult acute interventions.

### IL-1 Blockade

Our team and others uncovered that the upregulation of IL-1 plays a key role in chorioamnionitis, and in associated neonatal ischemic brain injuries (54, 79, 82, 84, 94–97). Our preclinical studies, and others, showed that prenatal IL-1 blockade using IL-1Ra is protective against chorioamnionitis, associated FIRS, and subsequent brain injuries (73, 74, 106–108). Postnatal administration of IL-1Ra is also effective in alleviating mortality (from 40 to 18%) as well as morbidities arising from postnatal inflammatory-sensitized NAIS (82, 92, 94): 66% decrease of the core (cavitary lesion), and 54% decrease of the penumbra (rim of mild to moderately ischemic tissue lying between the core and the unaffected tissue), and preventing the loss of motor skills (73, 74). IL-1Ra is an already approved drug to treat chronic inflammatory conditions, including those affecting pregnant mothers and newborns (96). IL-1Ra is, among the various molecules interfering with the IL-1 signaling, the one which dominates its pharmacological field due to its: (i) blocking effect on both IL-1α and IL-1β; (ii) short 4–6 h half-life (blood levels falling within a few hours of treatment stoppage); (iii) multiple routes of administration; (iv) approval for several pediatric inflammatory conditions (1–10 mg/kg/24 h), knowing that the repurposing of well-studied drugs used in the pediatric population is a cost-effective and efficient strategy to identify new therapies for pediatric diseases; and (v) excellent safety record (absence of opportunistic infection; reversible increase of liver enzyme, decrease of polymorphonuclear cells, slight increase of infection, that are mostly observed in patients on chronic treatment) after more than 10 years of use in more than 150,000 patients (80). Altogether, this provides encouraging preclinical evidence in favor of the efficacy and feasibility of end-gestational or neonatal interventions using IL-1Ra.

### Hypothermia Therapy

Hypothermia therapy is now a mandatory standard of care for term newborns suffering for diffuse HI encephalopathy (109–111). However, cooling treatment is modestly effective and leaves 50% of the treated patients with major sequelae (109, 112). Besides, it is uncertain why HT is effective for some, but not all, human newborns. Clinical studies reported that HT might have less beneficial effects on newborns exposed to infection-inflammation plus HI, than those exposed to HI alone (113–115). Furthermore, evidence in favor of an anti-inflammatory role of HT within the newborn brain is limited and conflicted. Only a few clinical or preclinical models address this question. However, a well-established anti-inflammatory effect of HT is the down regulation of oxidative stress within the brain (91, 97, 116–118). It has also been reported that HT is neuroprotective by limiting apoptotic cascades in human term newborns (110, 119–121). The potential effect of HT on neuroinflammatory cytokines expression has been poorly investigated up to now in preclinical models as well as in term newborns. It has been recently shown that HT did not modulate inflammatory molecules, including IL-1β, TNF-α, IL-1Ra and MMP-9, on LPS plus HI-exposed pups (118). Other downregulating effect of HT within preclinical NAIS brains remained unclear. In the clinical settings, HT has not been tested yet in NAIS patients, even though it is feasible and possibly effective.

### Erythropoietin

Erythropoietin presents anti-inflammatory and neuroprotective properties mainly through dampening free radical release and neural cells apoptosis that have been well-established on preclinical models of neonatal brain infarcts (122, 123). Erythropoietin seems to be well-tolerated and neuroprotective against perinatal brain lesions of premature newborns: Benders et al. performed a study in 21 consecutive NAIS patients diagnosed by magnetic resonance imaging (MRI) using erythropoietin (1,000 IU/kg intravenously administered just after the diagnostic confirmation by MRI, and repeated at 24 and 48 h) (124).

There was no adverse effect on blood cells counts, or coagulation. The residual *versus* initial MRI injuries were quantitatively compared at 3 months of age between the erythropoietin-treated patients *versus* 10 untreated matched historical controls. The percentage of tissue loss within the ischemic area was not different between the treated *versus* untreated group. Hence, the effectiveness of erythropoietin in terms of neuroprotection in the NAIS context remains to be established.

## Which Clinical Research Priorities for NAIS?

### Pre-NAIS Neuroinflammatory-Oriented Research Avenues

Before considering human therapeutic trials aiming to prevent the occurrence of NAIS, it is mandatory to improve our ability to rapidly and efficiently detect prenatal, and immediately postnatal inflammation, and to correlate inflammatory profiles with the occurrence of NAIS. Hence, large scale case–control studies are a necessary prerequisite to further identify subpopulation(s) of newborns at risk of NAIS (28), by comparing pertinent profiles of expression of inflammatory and prothrombotic markers—reflecting acute activation instead of genetic predisposition—in the umbilical cord tissues and cord blood samples, between NAIS *versus* non NAIS patients. In addition, there is an urgent need to design efficient diagnostic tools adapted to the rapid and non-invasive diagnosis of chorioamnionitis and other maternofetal infectious/inflammatory diseases. In this line, MRI of the placenta, which has been shown to rapidly detect abnormal placental signals in a preclinical model of chorioamnionitis might be a promising diagnostic option (125). Based on these potential predictive biomarkers of NAIS, preventive anti-inflammatory strategies might then be tested.

### Post-NAIS Neuroinflammatory-Oriented Research Avenues

A low threshold for early head MRI should be applied in newborns presenting acute neurological symptoms compatible with neonatal stroke. Adapted and thorough protocol of parenchymal and angiographic imaging should be continuously updated and more systematically applied (126), as recently recommended (127). The acute activation of inflammatory and prothrombotic factors at the time of the NAIS is an important research avenue to improved our physiopathological knowledge, and provide much needed biomarkers of NAIS. This might be performed first in retrospect using immunoassay on dried neonatal blood, as previously described (128). IL-1 blockers, erythropoietin, and/or hypothermia seem to be the most promising avenues to be tested in multicentric randomized control studies in the aim to limit the extent of NAIS and/or to promote recovery. Some teams already carried out retrospective and prospective phase I–II studies addressing such therapeutic approaches (124, 129, 130).

## Author Contributions

SC and GS contributed to the design of the manuscript. AG, CG, MC, SC, and GS drafted the manuscript. All the authors contributed to the editing of the manuscript.

## Conflict of Interest Statement

The authors declare that the research was conducted in the absence of any commercial or financial relationships that could be construed as a potential conflict of interest.

## Abbreviations

AI, autoimmune; Apaf-1, apoptotic protease activating factor-1; AS, Apgar score; BBB, blood–brain barrier; CCL, C-C chemokine ligand; CD, cluster of differentiation; CI, confidence interval; CRP, C-reactive protein; CXCL, C-X-C chemokine ligand; Cyt-c, cytochrome-c; DAMP, damage-associated molecular patterns; Drp1, dynamin-related protein 1; FADD, Fas-associated death domain; Fas-L, Fas ligand; FHR, fetal heart rate; FIRS, fetal inflammatory response syndrome; GA, gestational age; HI, hypoxia–ischemia; HT, hypothermia therapy; ICAM, intracellular adhesion molecule; IL, interleukin; IL-1Ra, interleukin-1 receptor antagonist; IL-1RacP, interleukin-1 receptor accessory protein; iNOS, inducible nitric oxide synthase; IUGR, intrauterine growth restriction; LPS, lipopolysaccharide; MAPK, mitogen-activated protein kinases; MCA, middle cerebral artery; MLKL, mixed lineage kinase domain-like; vWF, von Willebrand factor.
